# Supplementary material for: Life cycle comparison of industrial-scale lithium-ion battery recycling and mining supply chains
Source: Nat Commun. 2025 Jan 24;16:988. doi: 10.1038/s41467-025-56063-x (PMC11761346; doi:10.1038/s41467-025-56063-x)
Supplement: Supplementary file 1 — Supplementary Information [file 41467_2025_56063_MOESM1_ESM.pdf]

Supplementary Information

Life cycle comparison of industrial-scale lithium-ion battery recycling and mining supply chains

Michael L. Machala[1,2,#], Xi Chen[3,4,#,*], Samantha P. Bunke[3,#], Gregory Forbes[1], Akarys Yegizbay[5], Jacques A. de Chalendar[1], Inês L. Azevedo[1,2], Sally Benson [1,2], William A. Tarpeh[2,3,*]

[1]Department of Energy Science & Engineering, Stanford University, Stanford, California, 94305, United States

[2]Precourt Institute for Energy, Stanford University, Stanford, California 94305, United States

[3]Department of Chemical Engineering, Stanford University, Stanford, California 94305, United States

[4]School of Energy and Environment, City University of Hong Kong, Hong Kong SAR, China

[5]Department of Physics, Kenyon College, Gambier, Ohio 43022, United States

# These authors contributed equally

* Corresponding author emails: wtarpeh@stanford.edu, xche26@cityu.edu.hk

This Supplementary Information contains: Supplementary Note 1–5, Supplementary Tables 1–16, Supplementary Figures 1–6.

Supplementary Note 1. Drivers for recycling lithium-ion batteries

Projected LIB growth and supply

Historic and projected values for global lithium-ion battery (LIB) production by year are shown in **Supplementary Fig. 1**. In all projections, electric vehicles comprised the majority of LIB growth in the 2020s. The variation in reported values was due to data selection and availability, such as including plug-in hybrid electric vehicles in some projections while only considering fully electric vehicles in others. Projected major supply-demand deficits of critical materials were extracted from academic and industrial sources[1-7]. The years where the supply-demand gap fell below zero and did not recover to a positive value are shown in **Supplementary Fig. 1** for Li, Ni, Co, and Cu. The range of deficit years showed the supply and demand uncertainty of these critical elements, while the nearness of these years to the publication date of this manuscript highlighted the immediacy of supply risk and deficit concerns.

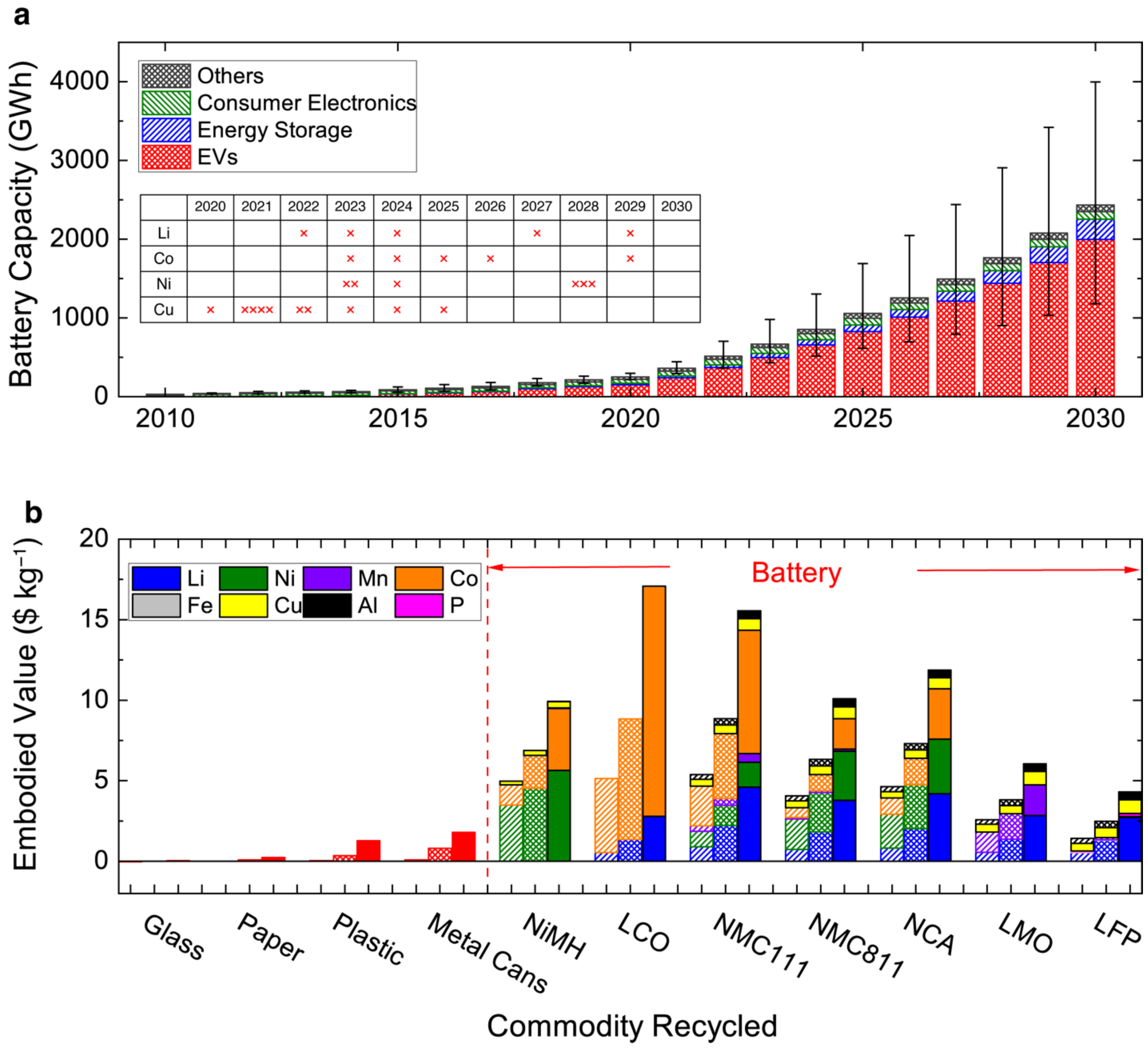

Supplementary Fig. 1 Battery capacity projections and embodied value of recycled materials.| a, Projected global demand of lithium-ion batteries in representative industries including electric vehicles (EVs), energy storage, consumers electronics. The uncertainty bars indicate the range of projections by different reports, highlighting varying optimism in supply and production capacity, along with methods of quantification, where hybrid or plug-in EVs may not be included in some analyses. Inset presents a compilation of industry report projections of when global supply-demand gaps will become negative and will not recover to positive values, running a supply deficit. Each "×" denotes a literature estimate[8-11]. **b,** composition breakdown of the embodied value of each commodity. Glass, paper, plastic, and metal cans were an average of three to five major sub-categories. The center bar for each commodity is the average value adjusted for inflation between January 2018 and December 2021 and in December 2021 USD [1-4,6,7,12,13]. The side bars represent the 90% confidence interval of those commodity values. Notably, all batteries chemistries represent 27-kWh battery packs with breakdowns provided by GREET 2021. LCO is included because it is a common battery in consumer electronics, although it is not a common EV battery material[14-16].

Embodied value of recycled materials

Economic value ranges for commonly recycled commodities (i.e., glass, paper, plastic, and metal cans) in **Fig. 1a** were based on historic monthly values in the U.S. from January 2018 to December 2021 considering inflation[14]. 90% confidence intervals, represented by uncertainty bars, showed variation in pricing not only based on time, but also based on commodity variety in each category. Because their constituent elements can be reused indefinitely, metal cans experienced recycling rates between 55–70%, whereas single-use plastic products were much lower at 3–30%. Fundamentally, carbon atoms have minimal intrinsic economic value due to abundance and the degradation of carbon-carbon bonds over multiple recycles.

Historic monthly LIB-relevant elemental value data were extracted from databases[14], and weighted by the composition of EV 27 kWh battery packs provided by GREET[17]. Values were also adjusted for inflation based on December 2021 USD. The relative composition of cathode powder in smartphone batteries was obtained from literature[18], and used to estimate the composition of an LCO-based smartphone battery. In both cases, the entire mass of the battery or battery pack was not included in reported recoverable value. The reported values were weighted by their proportion per kg of battery or battery pack. As such, additional recoverable materials (i.e., graphite, battery management system components) may still add additional economic value but were not considered here. Note that, by March 2022, prices for many critical elements were near or exceeding their 90% confidence maximum, while lithium carbonate prices had nearly doubled.

Supplementary Note 2. Emissions and water embodied in electricity consumption

The year 2021 was chosen as the reference year for electricity consumption. For each environmental impact category (energy, criteria air pollutants, and water), environmental impact conversion factors were used to calculate total impacts of electricity in each balancing area based on the composition of different electricity sources (e.g., natural gas, solar, hydroelectric). Total

electricity production and consumption in each balancing area, as well as exchanges with other balancing areas, were then used to compute the environmental impacts embodied in electricity consumption for each balancing area. This calculation generalized an approach that was previously developed for CO_2, SO_2 and NO_x[19-21], and incorporated additional data sources for electricity-embodied water[22] and criteria air pollutants (SO_x, $PM_{2.5}$, PM_{10}, CO, CH_4, N_2O)[22,23] Results of environmental impacts are summarized as an electricity life cycle inventory in **Supplementary Table 1**, and the original data sources are detailed in the **Supplementary Data File (spreadsheet)**. Reference years of data sources are listed in **Supplementary Table 2**.

Supplementary Table 1. Environmental impacts of circular refinement steps using different electricity sources for producing one kg of NCA-eq battery-grade materials ($LiNi_{0.80}Co_{0.15}Al_{0.05}O_2$). Circular refinement included reductive calcination (RC), mechanical (Me), and hydrometallurgical (Hy) steps, dealing with the production scrap and energized batteries. Note that the RC step was only required for energized batteries. Power sources were from Nevada renewable energy tariff (NV*), Bonneville Power Administration (BPAT), California Independent System Operator (CISO), and Western Area Power Administration–Colorado-Missouri (WACM)[19,20]. Data presented here are visualized in **Fig. 5b** in the main manuscript.

**Power sources**	**Feedstock/ Product**	**Step**	**Energy ($MJ\ kg^{-1}$)**	**Criteria Air Pollutant Emission**									**Water ($L\ kg^{-1}$)**
				**CO_2 ($kg\ kg^{-1}$)**	**CH_4 ($g\ kg^{-1}$)**	**N_2O ($mg\ kg^{-1}$)**	**CO ($g\ kg^{-1}$)**	**NO_x ($g\ kg^{-1}$)**	**SO_x ($g\ kg^{-1}$)**	**PM_{10} ($g\ kg^{-1}$)**	**$PM_{2.5}$ ($g\ kg^{-1}$)**	**CO_2-eq ($kg\ kg^{-1}$)**	
NV*	Recycled scrap/ Mixed sulfate	Me	1.67	0.02	0	0	0	0	0	0	0	0.02	4.64
		Hy	20.31	0.91	0.91	77.24	0.27	0.11	3.40	0.07	0.05	0.96	42.35
		Total	21.98	0.93	0.91	77.24	0.27	0.11	3.40	0.07	0.05	0.98	46.99
	Recycled battery/ Mixed sulfate	RC	2.45	0.24	0.05	19.13	0.03	0.02	0.01	0.01	0.008	0.25	6.38
		Me	3.18	0.03	0	0	0	0	0	0	0	0.03	8.83
		Hy	38.73	1.55	0.50	1.55	0.30	0.19	6.80	0.09	0.07	1.56	104.14
		Total	44.36	1.82	0.55	20.69	0.33	0.21	6.81	0.10	0.078	1.84	119.35
BPAT	Recycled scrap/ Mixed sulfate	Me	1.67	0.04	0.003	0.43	0.01	0.02	0.02	0.002	0.002	0.04	3.26
		Hy	20.31	1.13	0.93	80.74	0.33	0.26	3.53	0.09	0.07	1.17	30.97
		Total	21.98	1.17	0.94	81.17	0.34	0.28	3.55	0.09	0.07	1.22	34.22
	Recycled battery/ Mixed sulfate	RC	2.45	0.27	0.05	19.67	0.04	0.04	0.03	0.01	0.01	0.28	4.66
		Me	3.18	0.08	0.005	0.81	0.01	0.03	0.03	0.004	0.004	0.08	6.20
		Hy	38.73	2.09	0.56	10.48	0.50	0.56	7.14	0.13	0.11	2.11	75.13
		Total	44.36	2.45	0.62	30.95	0.52	0.64	7.20	0.15	0.11	2.48	85.99

Supplementary Table 1. Continued.

Power sources	Feedstock/ Product	Step	Energy ($MJ\ kg^{-1}$)	Criteria Air Pollutant Emission									Water ($L\ kg^{-1}$)
				CO_2 ($kg\ kg^{-1}$)	CH_4 ($g\ kg^{-1}$)	N_2O ($mg\ kg^{-1}$)	CO ($g\ kg^{-1}$)	NO_x ($g\ kg^{-1}$)	SO_x ($g\ kg^{-1}$)	PM_{10} ($g\ kg^{-1}$)	$PM_{2.5}$ ($g\ kg^{-1}$)	CO_2-eq ($kg\ kg^{-1}$)	
CISO	Recycled scrap/ Mixed sulfate	Me	1.67	0.13	0.005	0.74	0.02	0.04	0.02	0.006	0.006	0.13	0.76
		Hy	20.31	1.82	0.95	83.36	0.43	0.46	3.57	0.12	0.09	1.87	10.41
		Total	21.98	1.95	0.96	84.11	0.45	0.50	3.60	0.12	0.10	2.00	11.17
	Recycled battery/ Mixed sulfate	RC	2.45	0.38	0.06	20.06	0.06	0.07	0.04	0.02	0.01	0.39	1.54
		Me	3.18	0.24	0.009	1.41	0.04	0.08	0.04	0.01	0.01	0.24	1.45
		Hy	38.73	3.87	0.60	17.17	0.73	1.07	7.25	0.22	0.19	3.89	22.74
		Total	44.36	4.49	0.67	38.64	0.82	1.22	7.32	0.25	0.21	4.52	25.74
WACM	Recycled scrap/ Mixed sulfate	Me	1.67	0.31	0.05	7.07	0.09	0.22	0.29	0.02	0.02	0.32	1.25
		Hy	20.31	3.35	1.31	135.52	1.02	1.92	5.78	0.26	0.21	3.43	14.43
		Total	21.98	3.67	1.36	142.59	1.12	2.14	6.07	0.29	0.22	3.74	15.68
	Recycled battery/ Mixed sulfate	RC	2.45	0.61	0.11	27.96	0.15	0.29	0.37	0.04	0.03	0.62	2.15
		Me	3.18	0.60	0.09	13.45	0.18	0.42	0.55	0.04	0.04	0.60	2.38
		Hy	38.73	7.77	1.52	150.06	2.23	4.80	12.87	0.59	0.46	7.85	33.00
		Total	44.36	8.97	1.72	191.47	2.56	5.51	13.79	0.67	0.53	9.07	37.53

Supplementary Table 2. Summary of sources from different reference years and how they were used in this study. Data uses were categorized into three types to represent analysis result in the main manuscript (type I), literature data comparison in the main manuscript (type II), and supplementary information for additional reference (type III).

#	Supply Chain Step	Data Source for Presentation	Reference Year	Presentation of Data	Type of Use
1	Refinement	Redwood operational data for analyzing circular refinement	2021	Figs. 3–4	I
2		Environmental impacts of electricity for analyzing circular refinement	2021	Figs. 3–5	I
3		GREET model for analyzing conventional refinement	2021	Figs. 3–4	I
4		GREET model for analyzing conventional refinement	2023		III
5		Literature data of representative refinement technologies, for comparison	2019	Fig. 3	II
6	Extraction	For modeling	2021	Fig. 6	I
7	Transport	For modeling	2021	Fig. 6	I

Supplementary Note 3. Environmental Impact Differences

Output product: Lithium carbonate versus Lithium sulfate

Refinements analyzed in this study generated different output products (detailed in **Fig. 2** in the main manuscript). $NiSO_4$ and $CoSO_4$ were discrete salts; mixed hydroxide $(Ni,Co)(OH)_2$ and mixed metal sulfate $(Ni,Co)SO_4$ were products from the conventional and circular pathways, respectively. Discrete products were obtained from the mixed compounds with further separation processes, and the influences of different product formats on the environmental impacts of refinement procedure were analyzed in **Fig. 4** in the main manuscript.

Numerous previous studies used lithium carbonate (Li_2CO_3) as a reference compound; therefore, environmental impacts of both Li_2CO_3 and Li_2SO_4 were compared in this study for the reductive calcination pathway (RC+Me+Hy) and the hydrometallurgy pathway (Me+Hy) for energized LIBs. The results were presented in **Supplementary Figs. 2a–c** below, relative to Li_2SO_4, producing Li_2CO_3 exhibited higher energy consumption by 3.9–28.8%, CO_2-eq emissions by 6.4–29.3%, and water consumption by 2.1–21.9%.

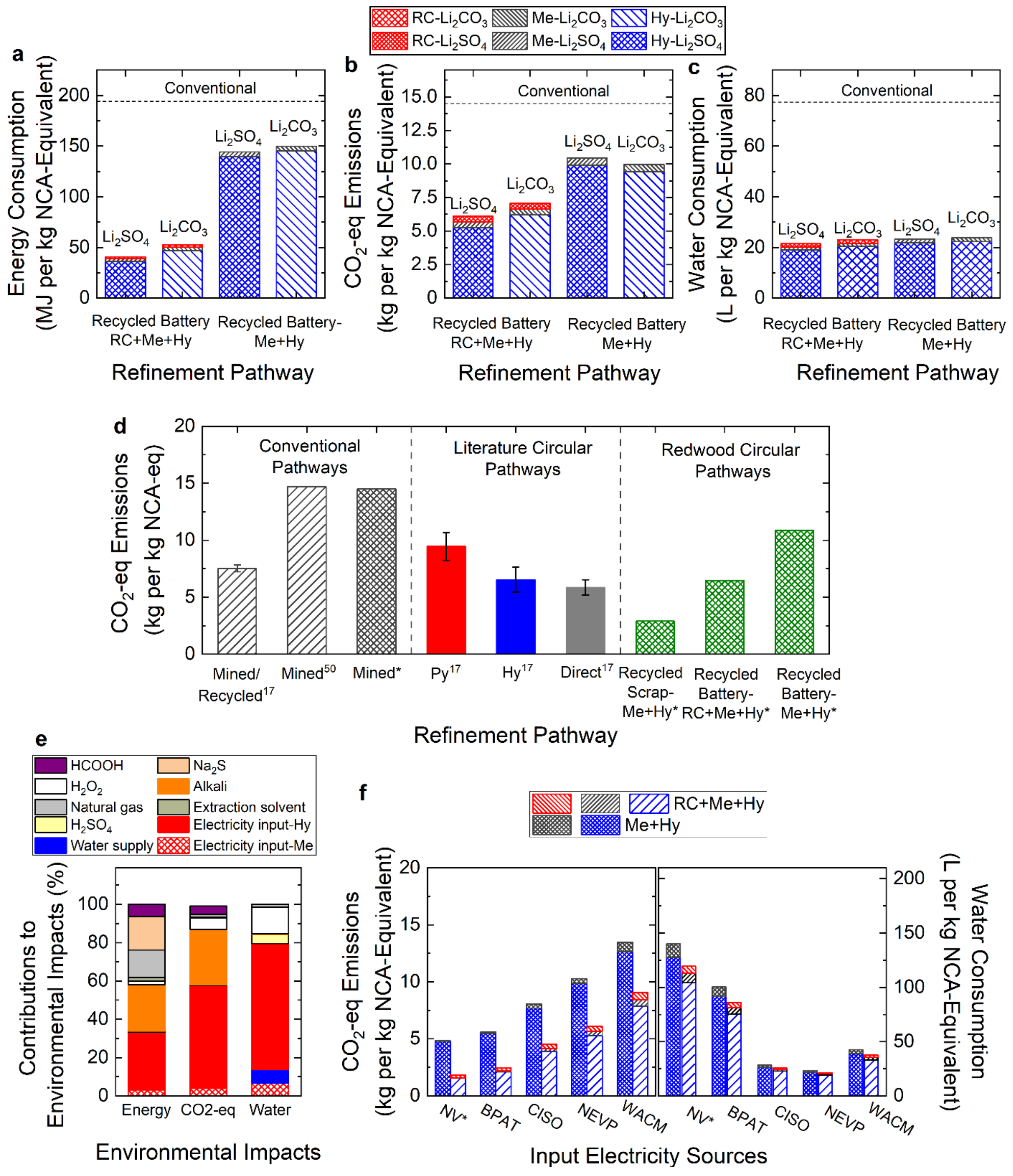

Supplementary Fig. 2 |Environmental impact comparison by refinement pathway. a, Comparisons of the environmental impacts including energy consumption, **b,** CO_2-eq emission, and **c,** water consumption of different refinement lithium products, Li_2SO_4 and Li_2CO_3, using Redwood (RC+Me+Hy) pathway and hydrometallurgy (Me+Hy) pathway. Both pathways refine energized batteries (recycled battery). Horizontal dash lines denote conventional mined pathways. **d,** Comparison of environmental impacts between refinement pathways in this study and data in literature[24,25]. In conventional pathways, data reported in literature were based on a combined sources including mined and recycled metals, and purely

mined sources. Circular pathways in literature all employ a single refinement technology, including pyrometallurgical (Py), hydrometallurgical (Hy), and direct recycling (direct). Data in this study and literature was denoted by "*" and superscripts of reference numbers, respectively. Data from the literature was normalized by the same functional unit in this study, and uncertainties were determined by combining two different battery form factors: pouch and cylindrical. **e,** Relative contributions of input energy, water, and consumables to the environmental metrics refining energized batteries by the hydrometallurgy (Me+Hy) pathway. **f,** Influences of balancing areas on CO_2-eq emissions and water consumption in Me+Hy and RC+Me+Hy pathways for recycled batteries.

Pathways: Explaining discrepancies in literature of similar pathways

Few previous studies have quantified the environmental impacts of recycling NCA LIBs. Results from a previous study assessing the CO_2-eq emissions of different prevailing circular refinement pathways and a conventional pathway were compared to the results from the current study[24] (visualized in **Supplementary Fig. 2d**). After normalizing the literature data by the same functional unit as in the current study, the previous study reported 7.53 ± 0.30 kg CO_2-eq per kg NCA-eq for the conventional refinement pathway, which was 48% lower than our result (14.5 kg per kg NCA-eq). This discrepancy is due to the inclusion of recycled elemental inputs for refinement into their analysis rather than considering only mined material[24]. Values of circular refinement pathways were 9.45 ± 1.21 CO_2-eq per kg, 6.53 ± 1.10 CO_2-eq per kg, and 5.86 ± 0.67 CO_2-eq per kg NCA-eq for pyrometallurgy, hydrometallurgy, and direct recycling, respectively.

The embodied CO_2-eq emissions were similar to the electricity used in the two studies (483 kg CO_2-eq per MWh in this study, and 510 CO_2-eq per MWh in the previous study). We attribute the different environmental impacts to the different chemical formulations of products in the two studies. While the previous study considered aqueous-phase metal salts as the final products, subsequent crystallization or precipitation of metal compounds will substantially increase the environmental impacts. For example, alkali consumables used for metal precipitation contributed 29% to the overall CO_2-eq emissions of the Me+Hy pathway (detailed in **Supplementary Fig. 2e**). The component contributions indicated that electricity was a principal contributor (57.4%) to CO_2-eq emissions in the Me+Hy pathway, implying that using electricity with lower CO_2-eq intensity can further decarbonize the prevailing circular refinement pathways.

In terms of decarbonization potential, electricity was identified as the dominant contributor to both Me+Hy (57%) and RC+Me+Hy (84%) CO_2-eq emissions of recycled batteries when utilizing NEVP electricity (**Supplementary Fig. 2e**). However, due to the greater contribution by electricity to the RC+Me+Hy pathway, it has a greater potential for decarbonizing based solely on electricity source versus Me+Hy (**Supplementary Fig. 2f**).

Supplementary Note 4. Environmental impacts of upstream material extraction

Two different methods for extracting battery-grade cathode materials were compared: mining natural ore and brine, and collecting end-of-life LIBs per the comparison of functionally equivalent process steps depicted in **Fig. 1c** of the main manuscript. The environmental impacts of mining were based on GREET 2021 and are summarized in **Supplementary Table 3**. Results based on GREET 2023 are summarized in **Supplementary Data**. Note that transportation of material between mining locations and subsequent refinement steps were often excluded in GREET, and production pathways incorporating transportation in between refining stages were modified by subtracting the associated environmental impacts of the transportation steps (**Supplementary Table 4**). Relevant values can also be found in **Supplementary Data**.

To estimate the emissions of collecting end-of-life energized LIBs from consumers to a refinement facility, a transportation logistics model was established for the state of California. California (CA) was chosen because it is the most populated US state (39.6 million, 2022) and has the highest number of electric vehicles, representing 42% of the entire country in 2020[26]. Existing collection facilities (CFs) were provided by the California Department of Resources Recycling and Recovery (CalRecycle)[26]. Two different LIB products were considered: end-of-life LCO-based smartphones and end-of-life NCA-based EV batteries. The assumed mass of transported smartphones and EV battery packs along with the mass of active cathode material is summarized in **Supplementary Table 5**.

In the first scenario, transport of smartphones from the census-block level to the nearest CF was used to estimate environmental impacts of extraction from consumers. Population in every census block was obtained from the U.S. Census Bureau[27]. One smartphone was assumed to be owned by each person in CA, and was recycled every three years. To determine the closest CF, k-means clustering was used for the allocation of census blocks to CFs based on minimizing the distance between a census block's centroid and a singular CF, for all census blocks. This process produced clusters of block groups whose LIB-embedded smartphones were allocated to the closest CF. The distance from the census blocks to the CFs was determined by the shortest route using Dijkstra's algorithm. The number of clusters k used in this algorithm was fixed as the total number of CFs. The relative size of a CF in the inset of **Fig. 6a** indicated the number of collected smartphones as compared to nearby CF clusters, and the smartphone collection areas were color-coded for distinction for each CF. Multiplying the resulting distances with the average mass of a smartphone yielded mass-distance products (i.e., kg × km), which were subsequently converted to environmental impacts using values for trucking (**Supplementary Table 6**). Note that the shortest-route value represented a lower bound of emissions.

In the second scenario, the number of registered zero-emission EVs in each CA county in 2021 was obtained from the California Energy Commission[28]. All EV batteries were assumed to be NCA-based and to reach end-of-life in 2021 (the reference year of this study). Because more than one CF existed in some counties, the CF that was closest to the centroid of that county was selected to represent the entire county. Additionally, because the distribution of EVs within each county was not known beyond the county-level, LIB collection was assumed to occur when an EV was driven to the CF for LIB extraction. The driving of an EV was considered a separate step in the use of an EV, and was excluded from the recycling lifetime; therefore, the environmental impacts associated with driving to a CF were assumed zero. Note that the removal of EV batteries from a vehicle requires additional investigations.

Supplementary Table 3. The gate-to-gate environmental impacts of conventional and circular LIB supply chains including energy consumption, criteria air pollutant emissions, and water consumption, for producing one kg of NCA-eq battery-grade materials ($LiNi_{0.80}Co_{0.15}Al_{0.05}O_2$). The conventional supply chain was based on the GREET 2021 model[17], starting from mined ores or brines (data based on GREET 2023 presented in **Supplementary Data** for reference). Circular refinement included reductive calcination (RC), mechanical (Me), and hydrometallurgical (Hy) steps, dealing with the recycled scrap and energized batteries, and was analyzed based on operating data in 2021. Note that the RC step was only required in recycled battery feedstocks. All circular data are based on power sources from Nevada Power Company (NEVP). The output products of Ni and Co can be both discrete salts ($NiSO_4$ and $CoSO_4$) and mixed compounds, $(Ni,Co)(OH)_2$ or $(Ni,Co)SO_4$. Other products considered for conventional refinement were Li_2CO_3 and Al_2O_3; the outputs produce from the circular supply chain were Li_2SO_4 and Al_2O_3 or $Al(OH)_3$. Li_2CO_3 was also analyzed as a product for Redwood Materials. "Method #" denotes the feedstock-pathway-products combination depicted in **Fig. 2** in the main manuscript. Data presented here are visualized in **Figs. 3–4** in the main manuscript, and **Supplementary Fig. 2** (note the comparison of Li_2SO_4 and Li_2CO_3 in **Supplementary Fig. 2a**).

**Supply chain**	**Method #**	**Feedstock/ Product**	**Element/ Step**	**Energy ($MJ\ kg^{-1}$)**	**Criteria Air Pollutant Emission**									**Water ($L\ kg^{-1}$)**
					**CO_2 ($kg\ kg^{-1}$)**	**CH_4 ($g\ kg^{-1}$)**	**N_2O ($mg\ kg^{-1}$)**	**CO ($g\ kg^{-1}$)**	**NO_x ($g\ kg^{-1}$)**	**SO_x ($g\ kg^{-1}$)**	**PM_{10} ($g\ kg^{-1}$)**	**$PM_{2.5}$ ($g\ kg^{-1}$)**	**CO_2-eq ($kg\ kg^{-1}$)**	
Conventional	(1)	Mined ores or brines/ Discrete salts	Li	41.61	3.85	5.61	23.23	2.84	3.63	4.52	1.03	0.68	4.00	13.61
			Ni	127.53	8.43	16.75	16.92	11.39	16.92	692.41	6.23	3.48	8.90	43.94
			Co	24.56	1.50	3.45	35.60	3.45	1.75	698.49	5.83	0.70	1.59	16.69
			Al	0.21	0.014	0.034	0.35	0.0089	0.016	0.020	0.012	0.0063	0.015	0.080
			Total	193.92	13.79	25.84	230.58	15.27	22.32	1395.4	13.10	4.87	14.50	77.32
	(2)	Mined ores or brines/ Mixed hydroxide	Li	41.61	3.85	5.61	23.23	2.84	3.63	4.52	1.03	0.68	4.00	13.61
			Ni-Co	302.53	22.35	36.19	381.28	29.41	61.28	423.75	5.30	4.13	23.27	58.52
			Al	0.21	0.014	0.034	0.35	0.0089	0.016	0.020	0.012	0.0063	0.015	0.080
			Total	344.36	26.11	41.83	404.86	32.26	64.92	428.29	6.34	4.82	27.28	72.21

Supplementary Table 3. Continued.

Supply chain	Method #	Feedstock/ Product	Element/ Step	Energy (MJ kg^{-1})	Criteria Air Pollutant Emission									Water (L kg^{-1})
					CO_2 (kg kg^{-1})	CH_4 (g kg^{-1})	N_2O (mg kg^{-1})	CO (g kg^{-1})	NO_x (g kg^{-1})	SO_x (g kg^{-1})	PM_{10} (g kg^{-1})	$PM_{2.5}$ (g kg^{-1})	CO_2-eq (kg kg^{-1})	
Circular	(3)	Recycled battery/ Discrete salts	Me	4.45	0.60	0	0	0.0001	0.0002	0.0002	0	0	0.60	1.41
			Hy	139.75	8.75	0.010	0.0030	0.0040	0.0035	0.013	0.00084	0.00064	9.89	21.78
			Total	143.2	9.71	0.010	0.0030	0.0041	0.0039	0.0132	0.00086	0.00066	10.85	21.64
	(4)	Recycled scrap/ Mixed sulfate	Me	1.67	0.21	0.01	2.15	0.04	0.09	0.07	0.01	0.01	0.21	0.59
			Hy	20.31	2.51	1.03	94.93	0.60	0.82	4.00	0.16	0.14	2.56	8.95
			Total	21.98	2.72	1.04	97.08	0.64	0.90	4.08	0.17	0.15	2.77	9.54
	(5)	Recycled battery/ Mixed sulfates	RC	2.45	0.45	0.07	21.82	0.08	0.13	0.11	0.02	0.02	0.46	1.32
			Me	3.18	0.40	0.03	4.08	0.08	0.16	0.14	0.02	0.02	0.40	1.12
			Hy	38.7	5.23	0.80	46.65	1.16	1.99	8.34	0.33	0.28	5.27	19.03
			Total	40.35	6.08	0.90	72.55	1.32	2.28	8.58	0.38	0.32	6.13	21.47

Supplementary Table 4. The estimated environmental impacts of transporting Li, Co, Ni, and Al concentrates of mined material in conventional supply chains normalized by the content of metal in transit (top table). The transportation impacts of aggregated LCO-based smartphones and NCA-based EV batteries were found in the bottom table from collection facilities to a recycling center positioned at the gravity point (i.e., center) of the California population. Data presented are visualized in **Fig. 6** in the main manuscript.

Conventional Material Transport

Material Transported	Energy Consumption (MJ per t metal)	CO_2-eq Emissions (kg per t metal)	Water Consumption (L per t metal)
Li concentrate	37,808	6,152	964
Co concentrate	70,149	6,445	1,727
Ni concentrate	44,755	5,355	1,073
Al concentrate	10,770	1,809	278

Circular Material Transport

Product Transported	Energy Consumption (MJ per t product)	CO_2-eq Emissions (kg per t product)	Water Consumption (L per t product)
Smartphone Collection*	20.5	1.00	0.490
Smartphone Transport	510	25.0	12.2
EV Battery Transport	515	25.3	12.3

*Smartphone collection is a material extraction step but included here for reference. Collection is modeled by the transportation resources required for battery collection from consumer census block and transport to a collection facility; it does not include other potential steps such as device disassembly for battery extraction from products.

Supplementary Table 5. Mass of lithium-ion battery embedded products considered in this analysis[17,18,29].

Battery Type	Element	Assumed Mass (kg)
LCO	LCO-battery embedded smartphone	0.118
	Mass of battery per smartphone	0.026
	LCO active material per kg of battery	0.162
NCA	End-of-life 27-kWh EV NCA battery pack	108
	NCA active material per NCA battery pack	37.4

Supplementary Table 6. Environmental impacts of fuels use by different modes of transport in the stages of "cradle-to-wheel", "cradle-to-tank", and "tank-to-wheel". Data were from references[17,30,31].

Transport Stage	Mode of Transport	Maritime	Rail	Road	Gasoline	Diesel	Residual Fuel Oil
Cradle-to-wheel	Energy Consumption (MJ per t km)	0.15	0.213	2.3	N/A	N/A	N/A
	CO_2-eq emissions (g per t km)	27.28	33.69	112.8	N/A	N/A	N/A
	Water Consumption (L per t km)	0.00396	0.00319	0.055	N/A	N/A	N/A
Cradle-to-tank	Energy Consumption (MJ per t km)	0.01	0.024	0.3	7.11	4.54	2.80
	CO_2-eq emissions (g per t km)	1.00	1.57	17.9	503	299	193
	Water Consumption (L per t km)	0.00396	0.00319	0.055	4.36	0.608	0.396
Tank-to-wheel	Energy Consumption (MJ per t km)	0.14	0.189	2.0	33.8	36.0	39.7
	CO_2-eq emissions (g per t km)	26.3	32.1	94.9	N/A	N/A	N/A
	Water Consumption (L per t km)	N/A	N/A	N/A	N/A	N/A	N/A

Supplementary Note 5. Environmental impacts of upstream material transport

Mined concentrate transport

A network model was used to determine the routes employed for transporting concentrated mined material from mining to refinement locations. The resulting mass-distance values were converted to environmental impacts using cradle-to-wheel fuel consumption data (**Supplementary Table 6**). For the critical LIB materials Li, Co, Ni, and Al, the contributions of material transported from mines to refineries were associated with and aggregated based on the countries in which they were extracted or refined (**Supplementary Tables 7–8**). The allocation of mined concentrates from mines to refineries was based on a weighted distribution because data detailing the transported amount and destination of material from every mine to every refinery was not accessible. Mined materials from each extraction country were often concentrated domestically and then transported overseas for refinement, with allocation weighted by the amount of refinement being performed relative to other refinery countries. The locations representing the mines and refineries were determined based on where most extractive and refinement activities take place (indicated by latitude and longitude coordinates). The locations were incorporated into the network model as the basis of origins and destinations of the routes for transportation logistics.

Specific transit routes were chosen for the network model, including road (i.e., by truck), rail (i.e., by train), and maritime (i.e., by shipping vessel) transport, exhaustive of the major routes available worldwide[32-35]. A portion of the network model and routes considered were presented in **Supplementary Fig. 4**. The ports associated with maritime transport connect all major sea ports to the closest rail junction. Extraction of Co in the Democratic Republic of the Congo (DRC) is a unique case, where logistics were limited to the road network for the transport of ores or brines from mines to a port[36]. The port in Beira, Mozambique is a major port for which mined Co from the DRC is transported for refining. The maritime network connects the port in Beira, Mozambique to the closest road junction to bridge the activity in the network model[37]. For a particular element, the shortest route utilizing all combinations between mines and refineries was calculated with Dijkstra's algorithm[38].

The normalized mass-based environmental impacts of LIB material concentrate transported, $\bar{e}^{\mathrm{m}}_{\mathrm{Mat},i}$, were calculated by **Supplementary equation** (1), where $e^{\mathrm{m}}_{\mathrm{Mat},i}$ is mass-based environmental impacts of the material i with a certain transport model (**Supplementary Table 6**), d_i is the segmental transport distance in the shortest-route model, n_{d} is the total segments along that route, and w_i is the element weight percent from the country of origin.

$$\bar{e}^{\mathrm{m}}_{\mathrm{Mat},i} = \frac{\sum_{i=1}^{n_{\mathrm{d}}} \left(e^{\mathrm{m}}_{\mathrm{Mat},i} d_i \right)}{w_i} \quad (1)$$

Environmental impacts with weighted distributions, $E_{\mathrm{Mat},i}$, for each element i were calculated to consider all routes for the three environmental impacts (**Supplementary equation** (2)):

$$E_{\mathrm{Mat},i} = \sum_{i=1}^{n_{\mathrm{d}}} \left(\bar{e}^{\mathrm{m}}_{\mathrm{Mat},i} m_i \right) \quad (2)$$

where m_i denotes the global percentage of an element considered for mined production or refinement production in 2021 (values summarized in **Supplementary Table 7–8**). Environmental impacts of embodied element are in **Supplementary Table 4**. Lastly, these values of $E_{\mathrm{Mat},i}$ were converted to LCO-eq and NCA-eq bases for comparison in **Fig. 6d–f** in the main manuscript.

Supplementary Table 7. Mine and mine cluster country with latitude and longitude used in analysis, mine capacity, and element weight percentage, for Li[39], Co[40], Ni[41], and Al[42]. Standard quantity was as reported and scaled quantity includes locations considered in the study scaled to 100%. References for all mine locations and assumed weight percentages of mined concentrates were obtained from the public repository: https://osf.io/zvame/?view_only=8fd188cde196485bbe625b217819242a.

Element Type	Mine Location	Latitude-Longitude Coordinates for Analysis	Mine Capacity (% of total / scaled to 100%)	Mine Capacity (standard quantity / scaled by total)	Element Content (wt. % of element in transported concentrate)
**Li**	Australia	(−33.85, 116.06)	52.3% / 52.9%	45,000 / 45,529	3.7
	Chile	(−26.23, −69.12)	22.4% / 22.7%	19,300 / 19,527	18.8
	China	(36.62, 101.78)	12.6% / 12.7%	10,800 / 10,927	18.8
	Argentina	(−23.79, −66.76)	7.3% / 7.4%	6,300 / 6,374	18.5
	Brazil	(−20.06, −44.57)	2.8% / 2.8%	2,400 / 2,428	3.7
	Zimbabwe	(−17.37, 31.43)	1.4% / 1.4%	1,200 / 1,214	2.2
**Co**	Democratic Re-public of Congo	(−8.75, 26.41)	69.4% / 80.4%	100,000 / 115,811	9.5 (5.0–14.0)
	Russia	(69.35, 88.21)	4.4% / 5.1%	6,300 / 7,296	12.5
	Australia	(−28.77, 121.88)	4.0% / 4.6%	5,740 / 6,648	3.0
	Philippines	(9.76, 125.51)	3.5% / 4.1%	5,100 / 5,906	4.9
	Cuba	(20.66, −74.95)	2.6% / 3.1%	3,800 / 4,401	5.0
	Madagascar	(−18.95, 48.30)	2.4% / 2.7%	3,400 / 3,938	4.2
**Ni**	Indonesia	(−1.43, 121.45)	25.3% / 35.1%	606,000 / 843,179	8.45
	Philippines	(9.48, 125.80)	14.4% / 20.0%	344,915 / 479,909	5.11
	Russia	(58.45, 92.19)	11.3% / 15.8%	272,000 / 378,457	9.45
	New Caledonia	(−22.37, 166.87)	9.0% / 12.5%	216,225 / 378,457	7.89
	Canada	(46.83, −71.25)	7.3% / 10.2%	175,761 / 244,551	15.7
	China	(30.66, 104.07)	4.6% / 6.4%	110,000 / 153,052	9.48

Supplementary Table 7. Continued.

Element Type	Mine Location	Latitude-Longitude Coordinates for Analysis	Mine Capacity (% of total / scaled to 100%)	Mine Capacity (standard quantity / scaled by total)	Element Content (wt. % of element in transported concentrate)
	Australia	(−31.95, 115.86)	29.3% / 34.1%	105,000 / 122,117	15.0
	China	(23.64, 108.27)	19.6% / 22.7%	70,000 / 81,411	24.3
	Guinea	(10.37, −13.58)	18.7% / 21.8%	67,000 / 77,922	23.8
**Al**	Brazil	(−1.46, −48.50)	9.5% / 11.0%	34,000 / 39,543	21.2
	Indonesia	(3.95, 108.14)	4.7% / 5.5%	17,000 / 19,771	21.2
	Jamaica	(18.04, −77.51)	2.5% / 2.9%	9,020 / 10,490	23.8
	Kazakhstan	(52.27, 77.00)	1.6% / 1.9%	5,800 / 6,746	23.0

Supplementary Table 8. Refinery location (latitude and longitude) and refining capacity for Li, Co, Ni, and Al. Standard quantity was as reported by USGS and others; scaled quantity included locations considered in the study scaled to 100%. References for all refinery locations were itemized and placed in a public repository[43].

Element	Refining Location	Latitude-Longitude Coordinates for Analysis	Refining Capacity (% of total / scaled to 100%)	Refining Capacity (standard quantity / scaled by total)
**Li**	China	(28.68, 115.88)	61.0% / 61.0%	52,306 / 52,306
	Australia	(−31.95, −115.86)	18.0% / 18.0%	15,403 / 15,403
	United States	(35.18, −81.34)	11.0% / 11.0%	9,627 / 9,627
	Chile	(−23.65, −70.40)	10.0% / 10.0%	8,664 / 8,664
**Co**	China	(24.72, 114.95)	67.0% / 75.3%	96,480 / 108,404
	Finland	(63.84, 23.13)	10.0% / 11.2%	14,400 / 16,180
	Canada	(50.00, −85.00)	5.0% / 5.6%	7,200 / 8,090
	Norway	(58.15, 8.00)	4.0% / 4.5%	5,760 / 6,472
	Japan	(33.96, 133.31)	3.0% / 3.4%	4,320 / 4,854
**Ni**	China	(27.99, 120.70	23.3% / 23.3%	244,900 / 244,900
	Canada	(50.00, −85.00)	15.5% / 15.5%	163,200 / 163,200
	Russia	(69.35, 88.20)	15.0% / 15.0%	157,396 / 157,396
	Japan	(33.96, 133.31)	11.6% / 11.6%	121,750 / 121,750
	Australia	(−27.28, 120.55)	9.9% / 9.9%	103,900 / 103,900
	Norway	(58.15, 8.00)	8.2% / 8.2%	86,500 / 86,500
	Finland	(61.31, 22.14)	5.7% / 5.7%	59,700 / 59,700
	South Africa	(−25.65, 27.26)	4.6% / 4.6%	48,100 / 48,100
	Madagascar	(−18.86, 48.30)	3.4% / 3.4%	35,474 / 35,474
	New Caledonia	(−22.28, 167.02)	2.9% / 2.9%	30,875 / 30,875

Supplementary Table 8. Continued.

Element	Refining Location	Latitude-Longitude Coordinates for Analysis	Refining Capacity (% of total / scaled to 100%)	Refining Capacity (standard quantity / scaled by total)
	China	(36.07, 119.16)	54.5% / 63.1%	195,150 / 225,971
	Australia	(−32.82, 151.71)	15.2% / 17.6%	54,373 / 62,960
	Brazil	(−2.53, −4.30)	6.5% / 7.6%	23,418 / 27,116
**Al**	India	(21.85, 84.03)	6.5% / 7.6%	18,008 / 20,852
	Russia	(61.67, 50.82)	5.0% / 5.8%	7,429 / 8,602
	Jamaica	(17.96, −77.60)	2.1% / 1.9%	5,841 / 6,764
	Saudi Arabia	(27.49, 49.14)	1.4% / 1.6%	4,953 / 5,735

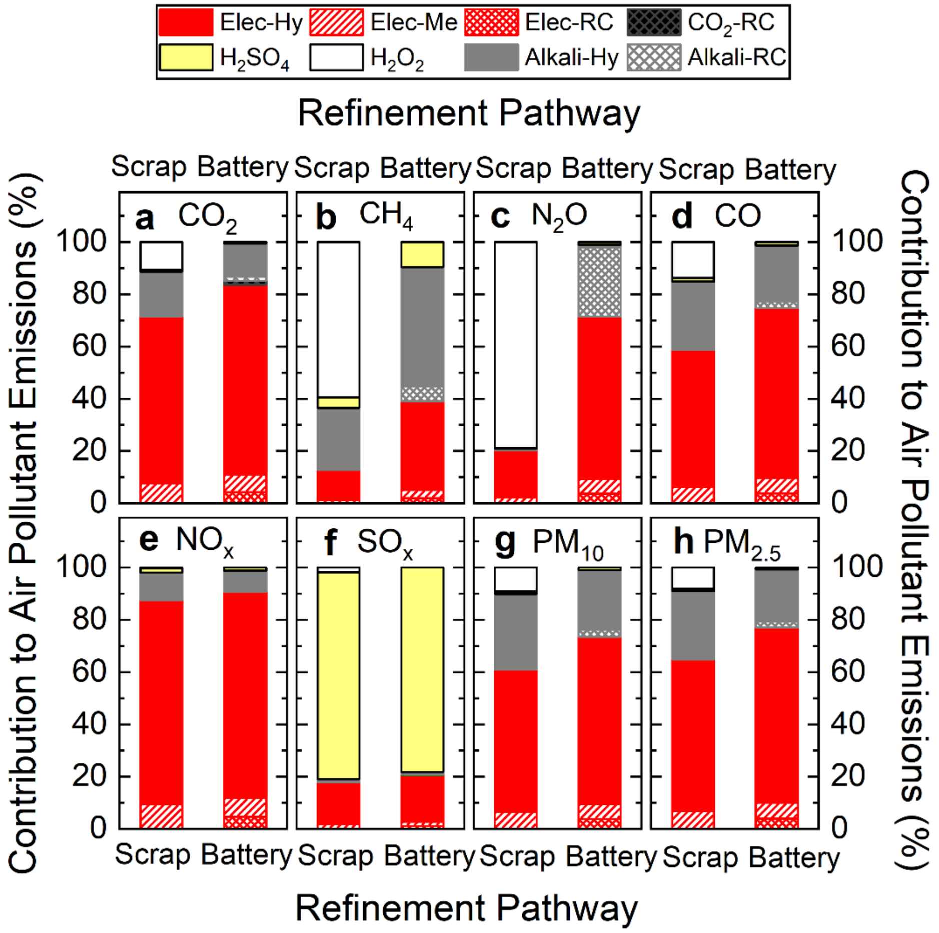

Supplementary Fig. 3 | Relative contributions to air pollutant emissions. **a,** CO_2, **b,** CH_4, **c,** N_2O, **d,** CO, **e,** NO_x, **f,** SO_x, **g,** PM_{10}, and **h,** $PM_{2.5}$, by lithium-ion battery circular refinement processes from production scraps and energized batteries based on Nevada electricity (NEVP). Note that CO_2-eq emissions presented in **Figs. 3–5** were calculated by summing the greenhouse gases multiplied by the corresponding 100-year global warming potential (GWP), including CO_2 (GWP = 1), CH_4 (GWP = 25), and N_2O (GWP = 298)[47].

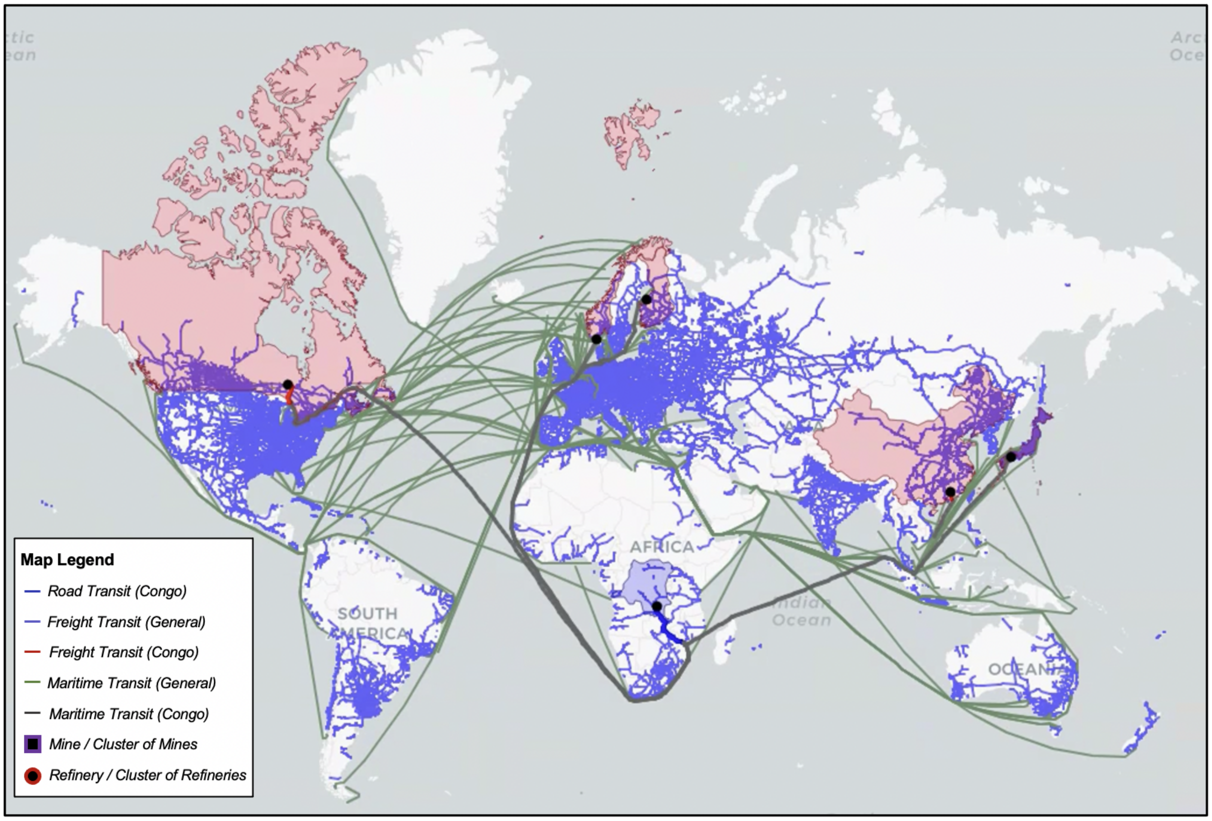

Supplementary Fig. 4 Network model for battery supply chains. | A portion of the network model used for transportation logistics showing major road, rail, and maritime routes. The overlapping transport of cobalt concentrate from the Democratic Republic of the Congo to major global refineries was also shown[32-35].

Formulations for life cycle impacts of fuel use by different transport mode

Life cycle inventories were developed for road, rail, and maritime modes of transport based on their use of fuel. The fuel used for each mode of transport was a composition of gasoline, diesel, and residual fuel oil, and the exact proportions (ϕ_j) were shown in **Supplementary Table 9**. Volumetric environmental impacts (e_i) for fuel *j* were acquired for "cradle-to-tank" and "tank-to-wheel" portions of the life cycle regarding energy consumption, CO_2-eq emissions, and water consumption (**Supplementary Table 6).** Efficiency, ξ_j, converts volumetric properties to mass-length basis (**Supplementary Table 9**). Environmental impacts of fuel for rail, road, and maritime modes of transport on a per-unit-mass-distance basis were calculated according to **Supplementary Equation** (3):

$$E_{\text{Fuel}} = \frac{\sum_{j=1}^{n}\left(e_{\text{F},j}^{\text{v}}\phi_j\right)}{\xi_j} \tag{3}$$

These results were summed for each fuel type proportionally for each mode of transport, producing the life cycle inventories for the entire life cycle (**Supplementary Table 6**).

Supplementary Table 9. Proportions of different fuel use, ϕ, and fuel efficiency ξ, by different transport modes. ξ were obtained from references[30,44-46]. Data presented were employed in **Supplementary equation** (3) for the visualization in **Fig. 6** in the main manuscript, and are quantified in **Supplementary Table 15**.

Transport Mode	Fuel	Proportion of Fuel Use, ϕ (%)	Fuel Efficiency, ξ (t km L^{-1})
Maritime	Gasoline	15.3	270.8
	Diesel	29.7	
	Residual Fuel Oil	55.1	
Rail	Gasoline	0	181.6
	Diesel	0	
	Residual Fuel Oil	100	
Road	Gasoline	9.9	18.7
	Diesel	89.4	
	Residual Fuel Oil	0	

Collected smartphones and EV battery packs

Environmental impacts of transportation of extracted LCO-based smartphones and NCA-based EV battery packs from CFs to the central recycling facility were calculated as a weighted distribution based on mass-distance. The mass and distance traveled were converted to energy consumption, CO_2-eq emissions, and water consumption using conversion factors from **Supplementary Table 6** and later normalized per kg of NCA-eq and LCO-eq precursor by the fractional percentage of active material contained in each transported product. Note that disassembly and extraction of battery cells from smartphones or EV battery packs prior to transport to a LIB recycling facility will alter calculated values.

Supplementary Table 10. Life cycle inventory for consumables used in circular refinement steps. Data for all consumables are generated using the GREET 2021 model and are normalized by 1 kg of each consumable[17]. Note that GREET 2023 provided no substantial change to the values listed below. Data of natural gas were obtained by averaging U.S. natural gas production from shale and conventional methods[44]. Total CO_2-eq were calculated by summing greenhouse gases multiplied by the corresponding global warming potential (GWP), with conversion factor global warming potentials (GWP) for 100 years: CO_2 (GWP = 1), CH_4 (GWP = 25), and N_2O (GWP = 298)[47].

**Consumable**	**Energy ($MJ\ kg^{-1}$)**	**Water ($L\ kg^{-1}$)**	**CO_2 ($kg\ kg^{-1}$)**	**CH_4 ($g\ kg^{-1}$)**	**N_2O ($g\ kg^{-1}$)**	**CO ($g\ kg^{-1}$)**	**NO_x ($g\ kg^{-1}$)**	**SO_x ($g\ kg^{-1}$)**	**PM_{10} ($g\ kg^{-1}$)**	**$PM_{2.5}$ ($g\ kg^{-1}$)**	**CO_2-eq ($kg\ kg^{-1}$)**
H_2SO_4	0.19	0.29	0.011	0.026	0.00023	0.0053	0.0088	20.01	0.0011	0.00067	0.011
HCl	31.62	5.20	1.82	4.66	0.39	0.98	1.57	0.86	0.16	0.11	1.94
HNO_3	12.05	5.06	0.67	2.10	4.77	1.51	1.45	0.21	0.027	0.025	2.15
H_2O_2	17.49	2.10	0.99	2.46	0.016	0.35	0.53	0.30	0.063	0.048	1.06
$Ca(OH)_2$	4.93	6.16	1.26	0.68	0.0012	0.46	0.26	0.14	0.14	0.10	1.28
NaOH	32.00	13.88	1.84	4.81	0.044	1.09	1.78	0.95	0.18	0.12	1.98
Natural gas	50.47	0.51	0.20	6.42	0.0014	0.51	0.57	0.48	0.017	0.015	0.36

Supplementary Table 11. Relative contribution of different inputs and consumables in the circular refinement processes (for scrap and energized battery feedstocks, "Method 4 and 5" in the main manuscript) to the environmental impacts, including energy consumption, CO_2-eq emissions, and water consumption. The data were based on the GREET 2021 model[17] and are visualized in **Fig. 5a** in the main manuscript. "N/A" denotes "not applicable."

**Environmental impact**	**Feedstock/ Product**	**Reductive Calcination**				**Mechanical**		**Hydrometallurgy**			
		**Electricity input (%)**	**Water input (%)**	**Alkali (%)**	**CO_2 emission (%)**	**Primary electricity (%)**	**Water input (%)**	**Primary electricity (%)**	**H_2SO_4 (%)**	**H_2O_2 (%)**	**Alkali (%)**
Energy consumption	Recycled scrap/ Mixed sulfate	N/A	N/A	N/A	N/A	6.16	N/A	50.75	6.28	10.72	23.50
	Recycled battery/ Mixed sulfate	4.70	N/A	0.82	N/A	7.16	N/A	79.09	1.44	N/A	6.79
CO_2-eq emissions	Recycled scrap/ Mixed sulfate	N/A	N/A	N/A	N/A	7.63	N/A	62.88	0.71	11.81	16.97
	Recycled battery/ Mixed sulfate	4.31	N/A	1.65	1.55	6.57	N/A	72.50	0.66	N/A	12.77
Water consumption	Recycled scrap/ Mixed sulfate	N/A	N/A	N/A	N/A	6.15	2.60	50.75	6.28	10.72	23.50
	Recycled battery/ Mixed sulfate	3.42	0.63	2.11	N/A	5.20	7.72	57.44	5.72	N/A	17.76

Supplementary Table 12. Life cycle inventory of embodied emission of air pollutants and water consumption of different electricity sources. Total CO_2-eq values were calculated by summing greenhouse gases multiplied by the corresponding 100-year global warming potential (GWP), including CO_2 (GWP = 1), CH_4 (GWP = 25), and N_2O (GWP = 298)[47]. Electricity sources were balancing grids from Bonneville Power Administration (BPAT), California Independent System Operator (CISO), Nevada Power Company (NEVP), Western Area Power Administration–Colorado-Missouri (WACM), and a Nevada renewable energy tariff (NV*) comprised of 85% geothermal, 10% solar, and 5% hydropower. Alternative power generated by hydraulic, nuclear, solar, natural gas (Ng), wind, coal, oil, biomass, geothermal, etc., were summarized in the lower sections. NEVP data listed are visualized in **Supplementary Fig. 3**. "N/A" denotes "not applicable" [19,20].

	Electricity source	Criteria Air Pollutant Emissions									Water ($L\ kWh^{-1}$)
		CO_2 ($kg\ MWh^{-1}$)	CH_4 ($kg\ MWh^{-1}$)	N_2O ($kg\ MWh^{-1}$)	CO ($kg\ MWh^{-1}$)	NO_x ($kg\ MWh^{-1}$)	SO_x ($kg\ MWh^{-1}$)	PM_{10} ($kg\ MWh^{-1}$)	$PM_{2.5}$ ($kg\ MWh^{-1}$)	CO_2-eq ($g\ MWh^{-1}$)	
Balancing Areas	BPAT	92.36	0.006	0.0010	0.016	0.038	0.035	0.0046	0.0040	92.78	7.02
	CISO	275.10	0.011	0.0016	0.044	0.09	0.046	0.013	0.012	275.10	1.65
	NEVP	453.77	0.031	0.0050	0.088	0.18	0.16	0.025	0.022	455.93	1.27
	WACM	674.84	0.105	0.015	0.20	0.47	0.62	0.051	0.040	681.99	2.70
	NV*	36.56	N/A	N/A	N/A	N/A	N/A	N/A	N/A	36.56	10.00
Electricity generation sources	Hydro	20.50	N/A	N/A	N/A	N/A	N/A	N/A	N/A	20.50	9.53
	Nuclear	13.00	N/A	N/A	N/A	N/A	N/A	N/A	N/A	13.00	2.18
	Solar	43.40	N/A	N/A	N/A	N/A	N/A	N/A	N/A	43.40	0.31
	Nat. gas	489.28	0.010	0.0016	0.067	0.11	0.0071	0.021	0.021	490	0.26
	Wind	13.00	N/A	N/A	N/A	N/A	N/A	N/A	N/A	13.00	0.01
	Coal	990.20	0.16	0.023	0.30	0.71	0.94	0.076	0.060	1001	1.55
	Oil	837.23	0.013	0.0082	0.51	3.70	2.29	0.24	0.217	840	0.33
	Biomass	31.30	0.11	0.060	1.18	0.68	0.049	0.073	0.069	52	5.50
	Geotherm	36.70	N/A	N/A	N/A	N/A	N/A	N/A	N/A	36.70	11.17
	Others	58.96	0.00087	0.00013	0.0057	0.010	0.00060	0.002	0.0018	59	7.34

Supplementary Table 13. Environmental impacts of the material extraction step for producing one kg of NCA-eq ($LiNi_{0.80}Co_{0.15}Al_{0.05}O_2$) mined natural material in the conventional supply chain. Impacts included energy consumption, criteria air pollutant emissions, and water consumption. The conventional extraction step was based on the GREET 2021 model[17]. Data presented are visualized in **Fig. 6d–f** in the main manuscript.

**Element**	**Energy (MJ/kg)**	**Criteria Air Pollutant Emissions**									**Water ($L\ kg^{-1}$)**
		**CO_2 ($kg\ kg^{-1}$)**	**CH_4 ($g\ kg^{-1}$)**	**N_2O ($mg\ kg^{-1}$)**	**CO ($g\ kg^{-1}$)**	**NO_x ($g\ kg^{-1}$)**	**SO_x ($g\ kg^{-1}$)**	**PM_{10} ($g\ kg^{-1}$)**	**$PM_{2.5}$ ($g\ kg^{-1}$)**	**CO_2-eq ($kg\ kg^{-1}$)**	
Li	8.25	0.58	0.74	7.56	0.33	1.22	0.047	0.057	0.048	0.60	5.35
Ni	26.06	1.95	2.59	97.78	1.31	6.53	3.93	0.52	0.40	2.00	7.77
Co	2.76	0.20	0.25	4.91	0.21	4.86	0.011	5.70	0.61	0.21	0.87
Al	2.0×10^{-3}	1.5×10^{-4}	1.8×10^{-4}	3.4×10^{-3}	1.1×10^{-4}	4.3×10^{-4}	8.1×10^{-5}	2.4×10^{-3}	1.2×10^{-3}	1.6×10^{-4}	0.016

Supplementary Table 14. Environmental impacts of material extraction and refinement steps in conventional supply chains, including energy consumption, criteria air pollutant emissions, and water consumption. The values were normalized by one kg of each element regardless of material type (e.g., ore type or elemental concentration). The conventional material extraction and refinement steps were based on the GREET 2021 model[17]. Note that values of lithium were weight averages of 45% brine- and 55% ore-based lithium production[48]. Refinement materials reference Li_2CO_3, $NiSO_4$, $CoSO_4$, Al_2O_3, and $MnSO_4$.

**Step**	**Element**	**Energy (MJ/kg)**	**Criteria Air Pollutant Emissions**									**Water ($L\ kg^{-1}$)**
			**CO_2 ($kg\ kg^{-1}$)**	**CH_4 ($g\ kg^{-1}$)**	**N_2O ($mg\ kg^{-1}$)**	**CO ($g\ kg^{-1}$)**	**NO_x ($g\ kg^{-1}$)**	**SO_x ($g\ kg^{-1}$)**	**PM_{10} ($g\ kg^{-1}$)**	**$PM_{2.5}$ ($g\ kg^{-1}$)**	**CO_2-eq ($kg\ kg^{-1}$)**	
Extraction	Li	114.78	8.07	10.24	105.16	4.53	17.02	0.66	0.79	0.66	8.40	9.41
	Ni	53.30	3.98	5.31	200.00	2.68	13.36	8.05	1.06	0.81	4.20	15.89
	Co	29.97	2.18	2.68	53.39	2.31	5.28	0.12	61.89	6.66	2.30	9.41
	Al	0.14	0.01	0.01	0.24	0.01	0.03	0.01	0.17	0.09	0.01	1.12
	Mn	23.11	1.34	2.76	23.45	0.86	1.11	0.73	5.29	2.65	1.40	6.73
	Cu	4.06	0.25	0.45	3.32	0.26	0.45	0.10	0.08	0.04	0.26	0.95
Refinement	Li	579.22	53.60	78.09	323.40	39.56	50.48	62.97	14.27	9.48	55.60	189.37
	Ni	260.87	17.24	34.26	350.60	23.30	34.62	1416.30	12.75	7.12	18.20	89.88
	Co	266.79	16.26	37.44	386.77	11.22	19.04	64.16	63.33	7.60	17.30	213.94
	Al	15.18	0.98	2.42	24.63	0.63	1.15	1.41	0.89	0.45	1.00	5.66
	Mn	1.08	0.86	0.11	1.78	7.3E-02	0.57	3.84	4.1×10^{-2}	3.6×10^{-2}	0.86	0.57
	Cu	32.25	1.99	4.68	42.26	1.78	2.23	140.28	0.21	0.14	2.10	5.26

Supplementary Table 15. Separated cradle-to-gate conventional and circular LIB supply steps showing energy consumption, greenhouse gas emissions, and water consumption for two different functional units: NCA-eq and LCO-eq cathode material salts. Conventional supply chain values reflected dominant global supply chains (excluding recycled feedstocks) extracted from GREET, with transport values modeled in this work. The circular supply chain represent recycling of NCA-based battery packs and LCO-based smartphones in California as described elsewhere in the study.

**Supply chain**	**Step**	**Energy Consumption**				**CO_2-eq Emission**				**Water Consumption**			
		**NCA**		**LCO**		**NCA**		**LCO**		**NCA**		**LCO**	
		Value (MJ per kg NCA-eq)	Percentage (%)	Value (kg per kg LCO-eq)	Percentage (%)	Value (kg per kg NCA-eq)	Percentage (%)	Value (kg per kg LCO-eq)	Percentage (%)	Value (L per kg NCA-eq)	Percentage (%)	Value (kg per kg LCO-eq)	Percentage (%)
Conventional	Extraction	37.1	14	26.2	10	2.85	14	1.96	9	14.0	15	10.9	7
	Transport	31.2	12	44.9	16	3.68	17	4.32	21	0.757	1	1.11	1
	Refinement	194	74	202	74	14.5	69	14.4	70	77.3	84	142	92
	Total	262	100	273	100	21.0	100	20.6	100	92.1	100	154	100
Circular	Extraction	0	0	0.385	0	0	0	0.0189	0.2	0	0	0.00920	0
	Transport	1.49	3.5	9.58	8	0.0729	2.2	0.470	5	0.036	0	0.229	0
	Refinement	44.4	97	112	92	4.11	98	10.39	95	38.0	99.9	96.1	99.8
	Total	45.8	100	122	100	4.18	100	10.88	100	38.0	100	96.3	100

Supplementary Table 16. Total metal extraction mass of ores or brines from global mining activity for Li, Co, Ni, and Al in 2019[49].

Element	Total Mined and Refined (t)
Li	86,000
Co	144,000
Ni	2,400,000
Al	189,000,000

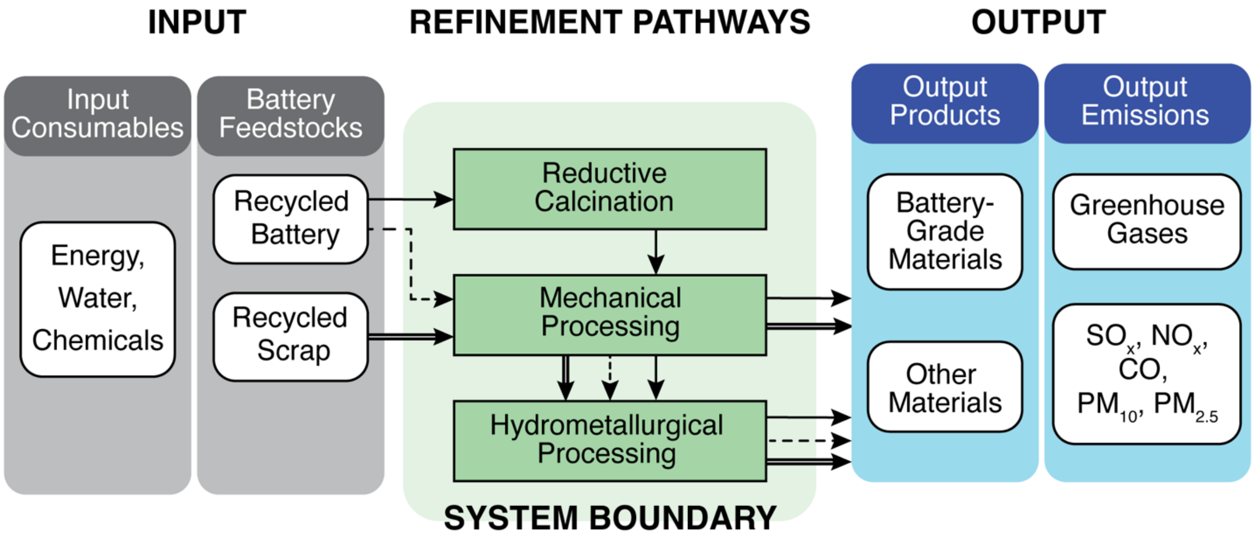

Supplementary Fig. 5 | General comparison of refinement pathways. Gate-to-gate circular refinement pathways at Redwood Materials for processing two lithium-ion battery (LIB) feedstocks: LIB production scrap (recycled scrap), and LIBs collected from consumers (recycled battery). Consumables including energy, water, and chemicals were inputs to the refinement pathways. Three refinement processes were employed in Redwood Materials including reductive calcination (RC), mechanical processing (Me), and hydrometallurgical processing/refinement (Hy). Recycled scrap was refined by a multi-step pathway employing Me and Hy (denoted by double-line arrows), while recycled battery were refined by an RC→Me→Hy multi-step pathway (denoted by solid single arrows) and a Hy-only pathway (denoted by dashed arrows). Output products were battery-grade materials and other materials (e.g., graphite), and output emissions included greenhouse gases and SO_x, NO_x, CO, PM_{10}, and $PM_{2.5}$. Note that in the system boundary only direct processes involved in refining pathways were analyzed, and no other site-wide operations (e.g., running office computers, lights) were considered.

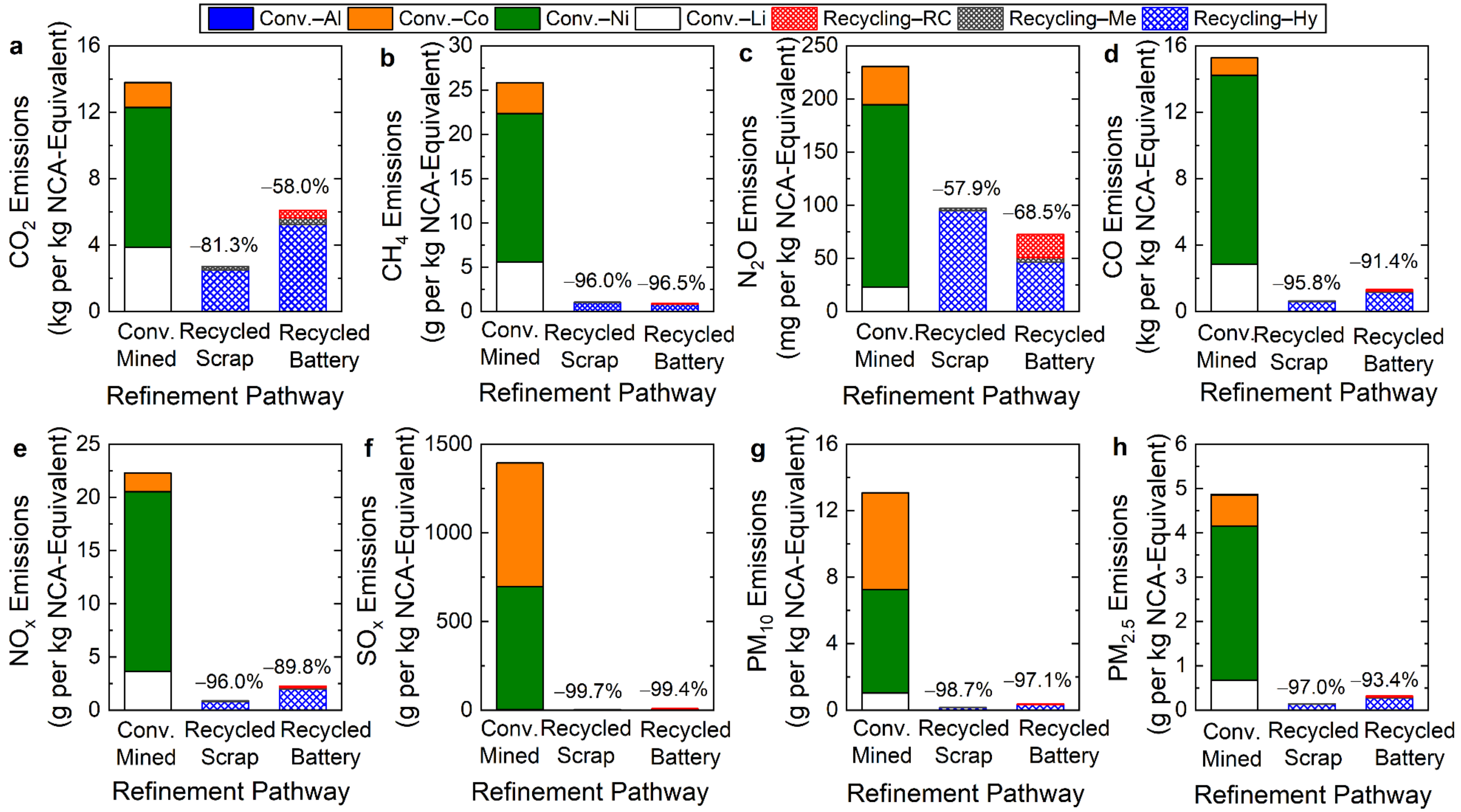

Supplementary Fig. 6 | Emissions of air pollutants. a, CO_2, **b,** CH_4, **c,** N_2O, **d,** CO, **e,** NO_x, **f,** SO_x, **g,** PM_{10}, and **h,** $PM_{2.5}$, by the mined conventional refinement and recycling processes from the scrap and energized batteries using Nevada energy (NEVP). Note that CO_2-eq emissions presented in **Fig. 3** in the main manuscript were calculated by summing the greenhouse gases multiplied by the corresponding 100-year global warming potential (GWP), including CO_2 (GWP = 1), CH_4 (GWP = 25), and N_2O (GWP = 298)[47].

Supplementary References

1 Xu, C. *et al.* Future material demand for automotive lithium-based batteries. *Commun. Mater.* **1**, 99 (2020).

2 Liu, B. *et al.* The impacts of critical metal shortage on China's electric vehicle industry development and countermeasure policies. *Energy* **248**, 123646 (2022).

3 Alves Dias, P., Blagoeva, D., Pavel, C. & Arvanitidis, N. Cobalt: demand-supply balances in the transition to electric mobility. 97710 (Publications Office of the European Union, 2018).

4 Valenta, R. K., Kemp, D., Owen, J. R., Corder, G. D. & Lèbre, É. Re-thinking complex orebodies: Consequences for the future world supply of copper. *J. Clean. Prod.* **220**, 816-826 (2019).

5 Battery recycling through extraction of metals from spent lithium-ion batteries
. (Syensqo, 2023).

6 Underwood, R. *et al.* Abundant Material Consumption Based on a Learning Curve for Photovoltaic toward Net–Zero Emissions by 2050. *Solar RRL* **7**, 2200705 (2023).

7 Fraser, J. *et al.* Study on future demand and supply security of nickel for electric vehicle batteries. Report No. 9276291393, (Publications Office of the European Union, 2021).

8 Pillot, C. The rechargeable battery market and main trends 2018-2023. (Avicenne Energy, 2018).

9 Li, D. Electric vehicles to drive massive battery demand: BNEF chart. (Bloomberg Law, 2021).

10 Cozzi, L. *et al.* World energy outlook 2020. *International Energy Agency: Paris, France* **2050** (2020).

11 Growth forecast for European EV market despite incentive impact. (EV Volumes, 2022).

12 Lombrana, L. M. & Farchy, J. A Million Tons of Copper is on the Way: It May not be Enough. *Bloomberg* (2019).

13 Mackenzie, W. Global Copper long-term outlook Q2 2019. *Wood Mackenzie* (2019).

14 Secondary materials pricing. (RecyclingMartket.net, 2022).

15 Zubi, G., Dufo-López, R., Carvalho, M. & Pasaoglu, G. The lithium-ion battery: State of the art and future perspectives. *Renew. Sustain. Energy Rev.* **89**, 292-308 (2018).

16 Chen, X., Shen, W., Vo, T. T., Cao, Z. & Kapoor, A. 230-235 (IEEE).

17 Wang, M. The greenhouse gases, regulated emissions, and energy use in transportation (GREET) 2021. *Center for Transportation Research, Argonne National Laboratory* (2021).

18 Gonçalves, M. C. A. *et al.* Chemical recycling of cell phone Li-ion batteries: application in environmental remediation. *Waste Manag.* **40**, 144-150 (2015).

19 de Chalendar, J. A., Taggart, J. & Benson, S. M. Tracking emissions in the US electricity system. *Proc. Natl. Acad. Sci. U.S.A.* **116**, 25497-25502 (2019).

20 de Chalendar, J. A. & Benson, S. M. A physics-informed data reconciliation framework for real-time electricity and emissions tracking. *Appl. Energy* **304**, 117761 (2021).

21 Nicholson, S. & Heath, G. Life Cycle Emissions Factors for Electricity Generation Technologies. (National Renewable Energy Laboratory, 2021).

22 Grubert, E. & Sanders, K. T. Water use in the United States energy system: a national assessment and unit process inventory of water consumption and withdrawals. *Environ. Sci. Technol.* **52**, 6695-6703 (2018).

23 Ou, L. & Cai, H. Update of Emission Factors of Greenhouse Gases and Criteria Air Pollutants, and Generation Efficiencies of the US Electricity Generation Sector. (Argonne National Lab.(ANL), Argonne, IL (United States), 2020).
24 Ciez, R. E. & Whitacre, J. F. Examining different recycling processes for lithium-ion batteries. *Nat. Sustain.* **2**, 148-156 (2019).
25 Crenna, E., Gauch, M., Widmer, R., Wäger, P. & Hischier, R. Towards more flexibility and transparency in life cycle inventories for Lithium-ion batteries. *Resour. Conserv. Recycl.* **170**, 105619 (2021).
26 Where to recycle: Map of public recycling locations. (CalRecycle, 2022).
27 United States Census Bureau Database. (U.S. Census Bureau, 2020).
28 Commission, C. E. Zero emission vehicle and infrastructure statistics. (California Energy Commission, 2022).
29 Roithner, C., Cencic, O. & Rechberger, H. Product design and recyclability: How statistical entropy can form a bridge between these concepts-A case study of a smartphone. *J. Clean. Prod.* **331**, 129971 (2022).
30 Davis, S. C. & Boundy, R. G. Transportation energy data book: Edition 39. (Oak Ridge National Lab.(ORNL), Oak Ridge, TN (United States), 2021).
31 Comer, B. in *International Council on Clean Transportation working paper* Vol. 3 (2019).
32 Center for International Earth Science Information, N. Global Roads Open Access Data Set (gROADS), v1 (1980–2010). *NASA Socioecon. Data Appl. Cent* (2010).
33 Exchange, T. H. D. Global railways (WFP SDI-T - Logistics Database) (The Humanitarian Data Exchange, 2017).
34 TIGER/Line Shapefile. (2021).
35 Aquaplot. (Aquaplot, 2021).
36 Maiotti, L. & Katz, B. Interconnected supply chains: due diligence challenges and opportunities sourcing cobalt and copper from the Democratic Republic of the Congo. (OECD Centre for Responsible Business Conduct, 2019).
37 Le Petit, Y. in *Transport and Environment* (2019).
38 Broumi, S., Bakal, A., Talea, M., Smarandache, F. & Vladareanu, L. 412-416 (IEEE, 2016).
39 USGS. Lithium statistics and information. (U.S. Geological Surveys, 2022).
40 USGS. Cobalt statistics and information. (2022).
41 USGS. Nickel statistics and information. (U.S. Geological Surveys, 2022).
42 USGS. Aluminum statistics and information. (U.S. Geological Surveys, 2022).
43 (2022).
44 Lopez Iii, C. E. *Optimizing energy power consumption of freight railroad bearings using experimental data*, The University of Texas Rio Grande Valley, (2020).
45 Baumel, P., Hurburgh, C. R. & Lee, T. Estimates of total fuel consumption in transporting grain from Iowa to major grain countries by alternatives modes and routes. *Iowa Grain Quality Initiative. Iowa* (2015).
46 Yacobucci, B. D. & Bamberger, R. Corporate Average Fuel Economy (CAFE): A Comparison of Selected Legislation in the 110th Congress. (2007).
47 2013 Revisions to the Greenhouse Gas Reporting Rule and Final Confidentiality Determinations for New or Substantially Revised Data Elements. Report No. 2013-27996, (Environmental Protection Agency, 2014).

48 Kelly, J., Dai, Q., Winjobi, O. Lithium pathway updates and additions in the GREET ® model. (Argonne National Laboratory, 2020).
49 Survey, U. S. G. *Mineral commodity summaries, 2021*. (Government Printing Office, 2021).
